# Supplementary material for: Structural insights into heterohexameric assembly of epilepsy-related ligand–receptor complex LGI1–ADAM22
Source: eLife. 2025 Jul 2;14:RP105918. doi: 10.7554/eLife.105918 (PMC12226019; doi:10.7554/eLife.105918)
Supplement: Figure 1—figure supplement 1—source data 2. [file elife-105918-fig1-figsupp1-data2.pdf]

a

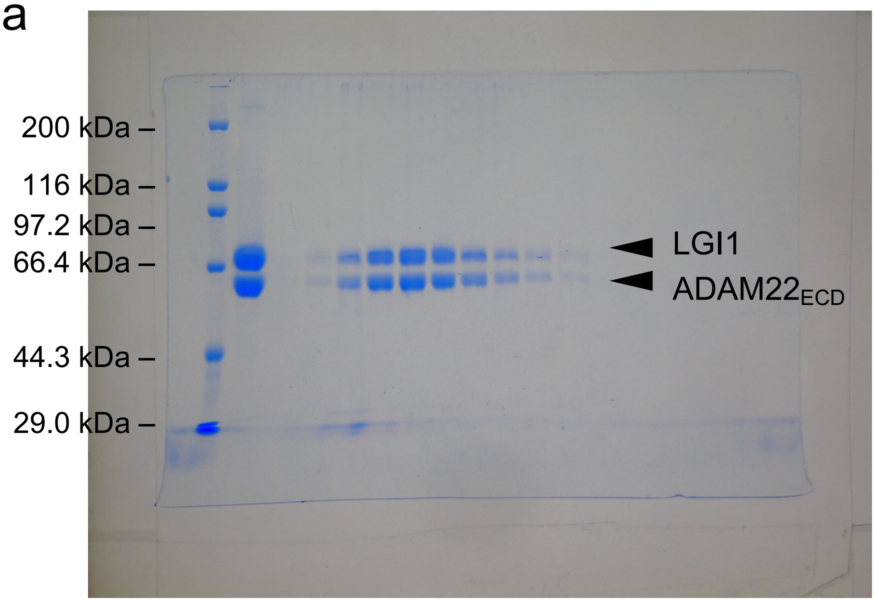

b

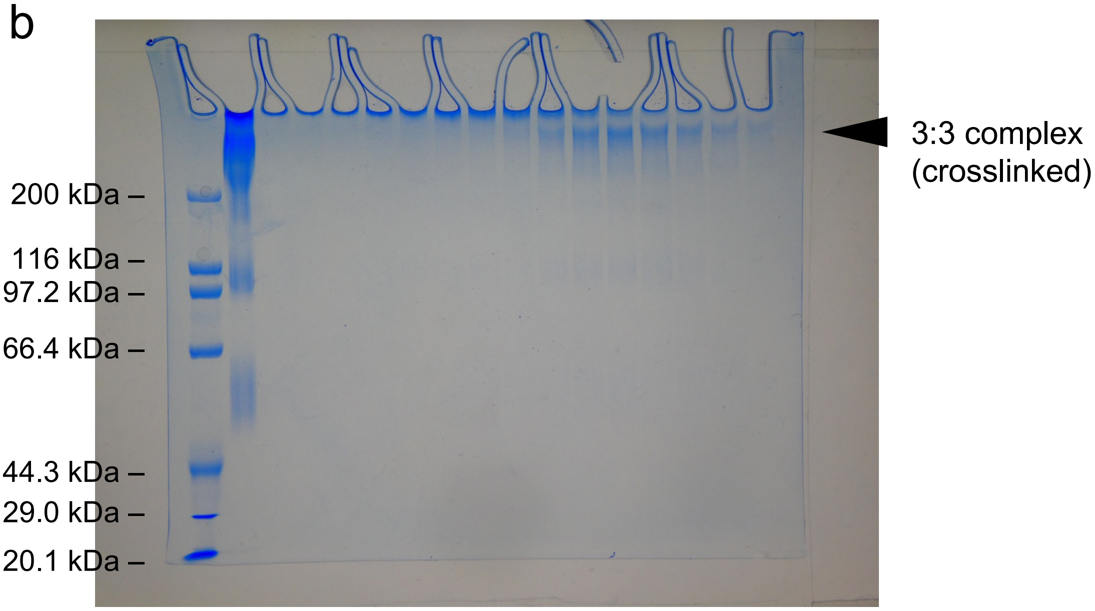

Figure 1–figure supplement 1–source data 2. Uncropped images of SDS-PAGE gels for Figure 1–figure supplement 1, indicating the relevant bands.

a Uncropped image of the CBB-stained SDS-PAGE gel displayed in Figure 1–figure supplement 1b.

b Uncropped image of the CBB-stained SDS-PAGE gel displayed in Figure 1–figure supplement 1d.
